# Body Temperature and Activity Adaptation of Short Photoperiod-Exposed Djungarian Hamsters (*Phodopus sungorus*): Timing, Traits, and Torpor

**DOI:** 10.3389/fphys.2021.626779

**Published:** 2021-07-07

**Authors:** Elena Haugg, Annika Herwig, Victoria Diedrich

**Affiliations:** Institute of Neurobiology, Ulm University, Ulm, Germany

**Keywords:** circadian rhythms, metabolism, phenotypes, radiotelemetry, Siberian hamster, spontaneous daily torpor

## Abstract

To survive the Siberian winter, Djungarian hamsters (*Phodopus sungorus*) adjust their behavior, morphology, and physiology to maintain energy balance. The reduction of body mass and the improvement of fur insulation are followed by the expression of spontaneous daily torpor, a state of reduced metabolism during the resting phase to save additional energy. Since these complex changes require time, the upcoming winter is anticipated *via* decreasing photoperiod. Yet, the extent of adaptation and torpor use is highly individual. In this study, adaptation was triggered by an artificially changed light regime under laboratory conditions with 20°C ambient temperature and food and water *ad libitum*. Two approaches analyzed data on weekly measured body mass and fur index as well as continuously recorded core body temperature and activity during: (1) the torpor period of 60 hamsters and (2) the entire adaptation period of 11 hamsters, aiming to identify parameters allowing (1) a better prediction of torpor expression in individuals during the torpor period as well as (2) an early estimation of the adaptation extent and torpor proneness. In approach 1, 46 torpor-expressing hamsters had a median torpor incidence of 0.3, covering the spectrum from no torpor to torpor every day within one representative week. Torpor use reduced the body temperature during both photo- and scotophase. Torpor was never expressed by 14 hamsters. They could be identified by a high, constant body temperature during the torpor period and a low body mass loss during adaptation to a short photoperiod. Already in the first week of short photoperiod, approach 2 revealed that the hamsters extended their activity over the prolonged scotophase, yet with reduced scotophase activity and body temperature. Over the entire adaptation period, scotophase activity and body temperature of the scoto- and photophases were further reduced, later accompanied by a body mass decline and winter fur development. Torpor was expressed by those hamsters with the most pronounced adaptations. These results provide insights into the preconditions and proximate stimuli of torpor expression. This knowledge will improve experimental planning and sampling for neuroendocrine and molecular research on torpor regulation and has the potential to facilitate acute torpor forecasting to eventually unravel torpor regulation processes.

## Introduction

The investigation of seasonal winter adaptation, including metabolic downstates like hibernation and daily torpor, in mammals has a long history (Jastroch et al., 2016), with promising implications for human life sciences, medicine, and manned spaceflight (Dave et al., 2012; Choukèr et al., 2019). Diverse species (bears, lemurs, marmots, bats, birds, squirrels, and dormice) in various environmental contexts (season, photophase, temperature, social context, food, and sleep) have been characterized *via* measurements of many different parameters at the whole animal level (behavior, morphology, and physiology) (Geiser, 2008). Over the years, the underlying regulatory mechanisms on the (neuro)endocrine and molecular genetic levels have become of increasing interest, and new analytical methods have opened. These methods either enable the measurement of classical *in vivo* parameters like activity, metabolic rate, body temperature, and body composition with higher resolution or the *in vitro* detection of new parameters like structure or transport proteins, enzymes, and RNA/DNA on the cellular and molecular levels. This rich repertoire offers countless possibilities to characterize the mechanisms of seasonal adaptation with a high degree of precision and standardization, improving comparability and reproducibility. However, the study of torpor regulatory mechanisms has remained complicated since torpor is a sensitive and difficult to anticipate physiological state. Hence, research would benefit from a method that better predicts adaptive strategies, including torpor.

Seasonal adaptations have been thoroughly investigated in the small rodent *Phodopus sungorus* (Pallas 1773). In long photoperiods, Djungarian hamsters are reproductively active and have a high body mass and a light brown summer fur. As soon as photophase length falls below 13 h per day (Hoffmann, 1982), the hamsters start to prepare for the harsh Siberian winter by reducing body mass as well as reproductive organs and by growing a well-insulating, white winter fur, to name only a few adaptation parameters (Scherbarth and Steinlechner, 2010; Cubuk et al., 2016). Approximately 3 months later, the morphological and physiological adaptations are largely completed, and the animals start to express spontaneous daily torpor. They use this metabolic downstate to save additional energy during their resting phase (photophase), while they are normothermic and active during the scotophase (Heldmaier and Steinlechner, 1981b; Diedrich et al., 2020). This complex temporal organization requires sensitivity to photoperiod length, based on daily light information as *zeitgeber*.

Most laboratory Djungarian hamsters are kept indoors in artificial light and subjected to abrupt changes of photoperiod. Independently of the time of year, they are bred in long photoperiod and transferred to short photoperiod to study the adaptation processes and spontaneous daily torpor. Although performed countless times since the beginnings of research in the 1970s, it has not been reported how the hamsters’ physiology reacts to this abrupt photoperiod change in the short and long terms.

Timing and the extent of seasonal adaptation in Djungarian hamsters can be highly individual within a hamster population. The morphology and physiology of respective adaptation phenotypes have been thoroughly characterized and categorized as responders, late responders, partial responders, or non-responders (Ruf et al., 1991, 1993; Przybylska et al., 2019), whereby responsiveness refers to the reaction to the change in photoperiod length. Non-responders retain a constant body mass and a brown summer fur and therefore do not express torpor. Responders show the already described morphological and physiological adaptations, yet with a high variability in timing and extent of single adaptive parameters. Even in responders, torpor expression is not obligatory under laboratory conditions. Although parameters like activity or food intake could be related to the incidence of torpor bouts (Ruf et al., 1991), it is still not possible to reliably extrapolate from certain adaptation parameters to later torpor expression.

In most institutes that contributed to the multiple studies on Djungarian hamsters during the last 40 years, torpor research comprises the *in vivo* characterization of responding hamsters and their torpor expression as well as a postmortem organ sampling for *in vitro* molecular analyses. The metabolic state of each hamster can be estimated from body temperature. Spontaneous daily torpor in Djungarian hamsters has been defined as a hypothermic core body temperature (*T*_b_) below 32°C (Ruf and Heldmaier, 1992; Ruf et al., 1993) for more than 30 min (Paul et al., 2005; Diedrich et al., 2015; Cubuk et al., 2017a, b). Single torpor bouts and the expression patterns can be further characterized using parameters like torpor incidence, torpor onset, minimal temperature during a torpor bout, torpor offset, as well as torpor duration (Kirsch et al., 1991; Ruf et al., 1993; Paul et al., 2004).

With continuous radiotelemetry measurements (Scribner and Wynne-Edwards, 1994; Schöttner et al., 2011; Jones et al., 2014), hypothermic hamsters with a body temperature below 32°C (HT, torpor) can be differentiated from normothermic hamsters (NT, no torpor) on the day of sampling (Bank et al., 2015). Usually, the sampling paradigm also comprises a temporal component allowing to differentiate circadian factors (Herwig et al., 2007; Bank et al., 2017; Cubuk et al., 2017a, b). This high degree of sampling standardization is of great value for the *in vitro* characterization of the molecular mechanisms underlying the different stages of torpor expression.

However, the sampling paradigm is based on each hamster’s acute torpor expression. The high interindividual variability in torpor incidence as well as the torpor onset or torpor bout duration might affect the desired equal distribution across sampling groups.

To define parameters in order to better predict torpor expression in individuals during the torpor period, the present study examined the existing body temperature and activity datasets of responders, which had been dedicated to molecular organ analyses on torpor regulation. In an additional approach, data were analyzed over the entire adaptation process to a short photoperiod, aiming at the early estimation of the adaptation extent and torpor proneness. Additionally, winter fur development and body mass reduction were determined over the course of adaptation in all animals. The phenotyping toward torpor incidence may help improve the *a priori* planning of experimental groups for a refined sampling with high standardization. Furthermore, the identification of potential predictors on general torpor capability would enable a preselection of torpor-prone hamsters early during adaptation in favor of animal reduction in future experiments.

## Materials and Methods

### Breeding and Housing

Djungarian hamsters (*P. sungorus*) were bred, raised, and kept at the Institute of Neurobiology, Ulm University, at 20 ± 1°C ambient temperature. Artificial light (150 lx) was provided 16 h per day in long photoperiod (LP) and 8 h per day in short photoperiod (SP). Additional constant red light (<5 lx) enabled animal handling during the scotophase. The hamsters were housed in Makrolon type III cages (26.5 cm × 42.5 cm × 18.0 cm) with wooden bedding and tissue as the nesting material. Tap water and food (Altromin hamster breeding diet 7014, Lage, Germany) were provided *ad libitum*. Additionally, cucumber, oak flakes, and sunflower seeds were fed once a week. The hamsters were bred in artificial long photoperiod by an outbred crossing scheme in accordance with the local ethics committee (35/9185.46-3) at Ulm University, Germany. During breeding, hamster pairs were provided with additional nesting material and a red transparent plastic house. Litters were weaned at an age of 3 weeks and housed in same-gender sibling groups. The hamsters were single housed since the age of 6 weeks. They were transferred to the short photoperiod earliest at 12 weeks of age. The expression of spontaneous daily torpor was expected during the torpor period after adaptation to the short photoperiod was largely completed.

### The Experiment

Between 2018 and 2020, 80 adult hamsters were adapted to SP with an average age of 4 months and sampled approximately 4 months later during the torpor period for molecular torpor research. The change of light regime from long to short photoperiod delayed the zeitgeber time 0 (ZT0) by 1 h and advanced the beginning of the scotophase by 7 h to favor the later realization of the described temporal sampling paradigm. The hamsters were approached daily by the caretaker between ZT07 and ZT08 for a visual check. Once a week, the animals were handled just before ZT08 by the experimenter to assess the progress of short photoperiod adaptation. Therefore, the relative body mass change (in percent) with respect to the absolute body mass of the last week in LP was monitored. In addition, the hamsters’ fur index was scored from 1 for a light brown summer fur to 6 for a dense white winter fur (Figala et al., 1973). During handling, wooden bedding and nesting material were refreshed every other week.

To measure body temperature and activity, a radiofrequency transmitter [Data Sciences International (DSI), Harvard Bioscience Inc., St. Paul, MN, United States] was implanted intraperitoneally under isoflurane anesthesia (2.5% and 1 ml/min for induction, 0.75–2.0% and 0.4 ml/min for maintenance) and carprofen analgesia (5 mg/kg, i.p.; Rimadyl, Zoetis Deutschland GmbH, Berlin, Germany). Recovery from surgery was supported by additional oat flakes, sunflower seeds, cucumber, and nesting material. Body mass, coat care, posture, and behavior were monitored daily for about 7 days. Experimental and surgical procedures were approved by the Regierungspräsidium Tübingen, Germany (1411).

Twenty hamsters, implanted during the torpor period, expressed spontaneous daily torpor within 2 weeks after implantation and were directly sacrificed. Data of the remaining 60 hamsters were used for this study, which required radiotelemetry data of 1 week after at least 1 week of recovery from surgery.

#### Approach 1: Torpor Behavior (*n* = 60)

The torpor behavior of 60 hamsters (41 males and 19 females) was analyzed using radiotelemetry data recorded during the torpor period after SP exposure for 15 ± 2 weeks. For animals that expressed torpor, the last week before sacrifice was chosen as the representative week of analysis. To favor a comparable adaptation state, the first possible week after 1 week of recovery from surgery was chosen as the week of analysis for hamsters that never expressed torpor during their individual total observation interval. Detailed background information on each hamster’s age when transferred to SP and the weeks spent in SP at implantation, observation, and sacrifice is listed in the Supplementary Table 1.

**FIGURE 1:**
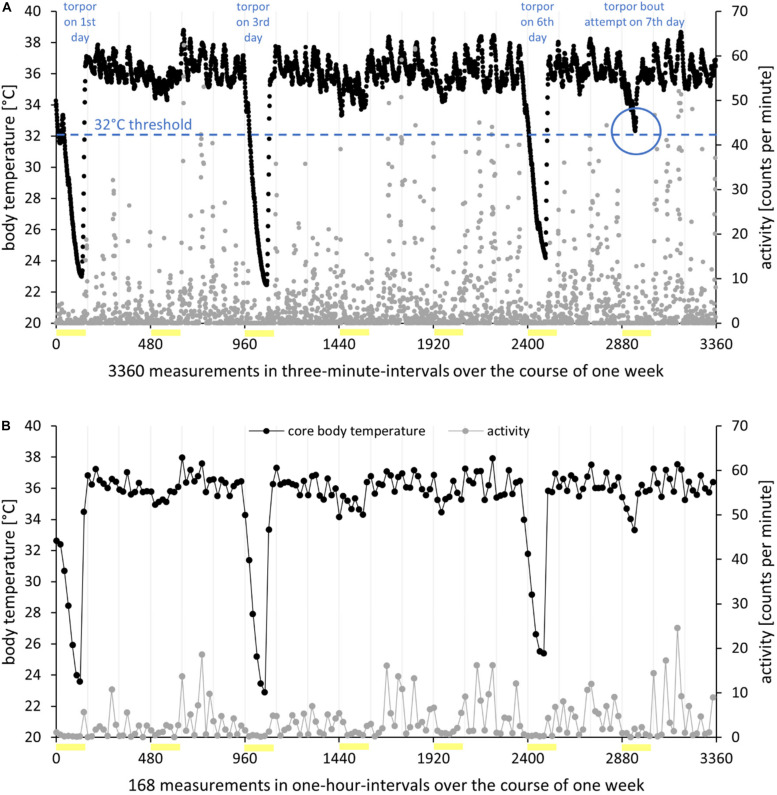
Processing of radiotelemetry data I. Body temperature (in degree Celsius, *black*) and activity (in counts per minute, *gray*) of hamster EH02-27 over the course of 1 week in the short photoperiod (L/D 8:16 h). Photophases are indicated as *yellow bars*. **(A)** High resolution. Real-time raw data recorded in 3-min intervals. Within the 7 days, the hamster expressed three torpor bouts per definition with body temperature below 32°C for at least 30 min, resulting in a torpor incidence of 0.43. On the last day of the analysis week, the hamster showed a torpor bout attempt. While the body temperature hardly changed from one measurement to the next, the activity data were highly variable. **(B)** Low resolution. Processing of raw data to mean values per hour resulted in a smoothing of the body temperature measurements; however, the activity data became more accessible.

Torpor incidence was calculated as the number of torpor bouts divided by the number of observation days, i.e., 7 days of the analysis week (Figure 1A). Furthermore, a decrease of body temperature to at least 33°C which did not result in a torpor bout per definition is referred to as a torpor bout attempt. The minimal body temperature is the lowest value during a torpor bout. Temporal values are given in hours and minutes after the beginning of the photophase at ZT0. Torpor onset was defined by the time point or ZT when a hamster reached a body temperature of 32°C first and consequently entered a torpor. Torpor offset was defined by the time point or ZT when the body temperature exceeded the threshold of 32°C first during the hamsters’ arousal from a torpor bout. Torpor duration was defined as the time between torpor onset and torpor offset (Ruf et al., 1993; Paul et al., 2005).

#### Approach 2: Change of Light Regime and Adaptation to Short Photoperiod (*n* = 11)

Eleven of the 60 hamsters analyzed in approach 1 (five females and six males, bred in 2020) had already been implanted in long photoperiod for long-term radiotelemetry measurements until sacrifice for molecular torpor research in SP14. Data acquisition started after one and a half weeks of surgical recovery and comprised 1 week in long photoperiod (referred to as LP baseline or SP00) and 13 weeks in short photoperiod (from SP01 to SP13). The development of body mass, fur index, body temperature, and locomotor activity over the course of SP adaptation was shown for individuals and the cohort to provide a visualization of variance within the cohort and statistical analyses of trends.

Data on relative body mass reduction and relative torpor incidence of 10 hamsters (2018 breed) have already been published in another research context (Piscitiello et al., 2021), while studies on molecular analyses of the hamsters’ tissues are in progress. Therefore, the radiotelemetry data of this study are not yet published for free use, but can be provided on request.

### Radiotelemetry

The radiotelemetry system (Dataquest ART^TM^ Bronze System, DSI, Harvard Bioscience Inc., St. Paul, MN, United States) comprised a pre-calibrated and silicone-coated TA11TA-F10 radiofrequency transmitter (PhysioTel^TM^, DSI, 1.1 cc volume, 1.6 g weight, 0.15°C measurement accuracy) implanted in the hamster’s peritoneal cavity, a receiver plate (RPC-1, DSI) underneath the hamsters’ home cage, a 20-channel Data Exchange Matrix (DEM, DSI), and a personal computer (Windows 7, 64-bit) outside the animal room. The raw data of core body temperature (in degree Celsius) and activity (counts per minute, cpm) were recorded in intervals of 3 min by the software “DataquestARTbronze” (2013). Body temperature was measured at the end of every 3-min interval. Activity was derived from the change of signal strength induced by the hamster, and therefore transmitter movement, relative to the receiver plate. This change was measured every 10 s and transformed by the system to cpm. At the end of every 3-min interval, an average activity value with the time-dependent unit cpm was recorded. An absolute activity could not be provided by the system.

### Data Processing

**FIGURE 2:**
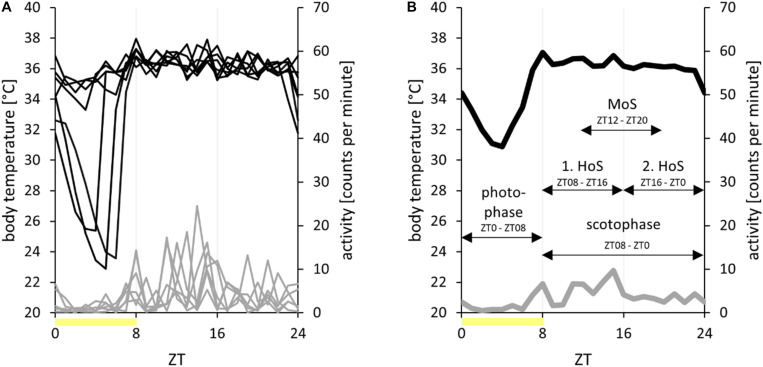
Processing of radiotelemetry data II. Data of body temperature (in degree Celsius, *black*) and activity (in counts per minute, *gray*) of hamster EH02-27 over the course of 1 week in the short photoperiod (L/D, 8:16 h) with the photophases indicated as *yellow bars*. **(A)** Overlay. The data of each of the 7 days of the analysis week are overlaid for visualization on an *x*-axis from zeitgeber time 0 (ZT0) to ZT24. **(B)** Time frames. The data were averaged per ZT and week to visualize the body temperature and activity patterns of hamster EH02-27 over 1 week. In this study, the data were averaged per time frame and week (*arrows*). The time frames photophase, scotophase, first half of scotophase (*1. HoS*), second half of scotophase (*2*. *HoS*), and middle of scotophase (*MoS*) were used. Please note that the mean values per time frame and their standard deviations have been directly calculated from the raw dataset, on which the analysis of torpor parameters was also based.

Raw data processing and graphical representation were performed with Microsoft Excel (Microsoft Office 365, 2016), unless otherwise stated. While the activity dataset remained raw, the body temperature dataset was corrected for measurement errors by deleting physiologically impossible temperatures below ambient temperature or above 42°C as well as values with unphysiological fluctuations of more than 0.5°C per 3-min interval (Heldmaier and Ruf, 1992; Figure 1A). On these corrected data, the analyses of torpor parameters and the standard deviation of the mean body temperature were based. For analyses on a larger scale, data were averaged per hour (Figure 1B), week (Figure 2A), and time frame (Figure 2B). This allowed for comparisons between time frames within 1 week of analysis (approach 1) and the development of body temperature and activity in steps of 1 week over the course of adaptation to the short photoperiod (approach 2).

### Time Frames

**FIGURE 3:**
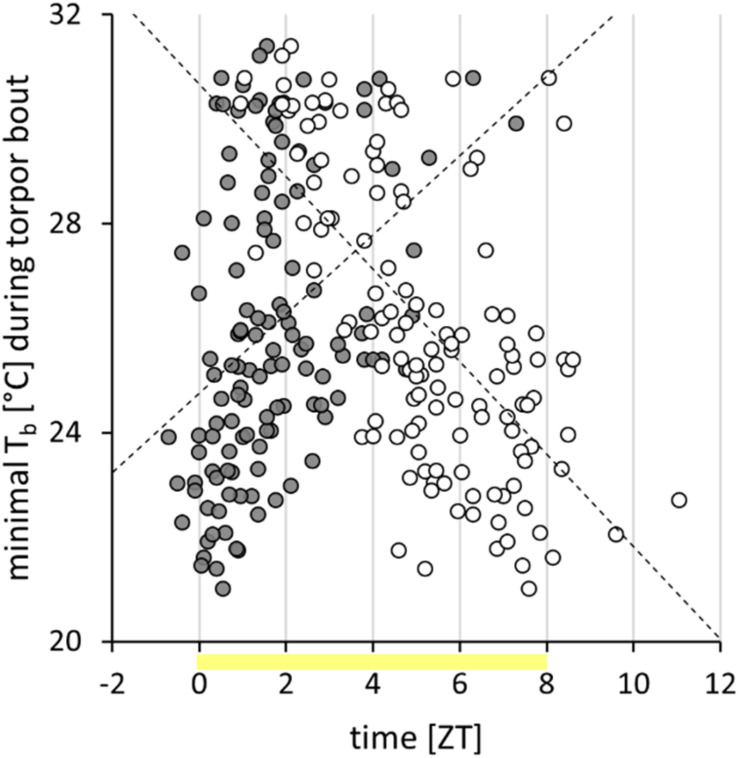
Torpor characteristics of approach 1. Within the torpor analysis week, 127 torpor bouts, performed by 42 Djungarian hamsters, were examined. Of each bout, the torpor onset (*gray circles*) and the torpor offset (*white circles*) are shown. The minimal body temperature (*T*_b_) during each torpor bout is positively correlated with its onset (*r* = 0.385, *p* < 0.05) and negatively correlated with its offset (*r* = -0.628, *p* < 0.05). Most bouts were performed within the photophase (*yellow*).

The total time frame accounted for the entire dataset of each analysis week. In SP, the photophase expands from ZT0 (including) to ZT08 (excluding), as shown in Figures 1–3. Accordingly, the scotophase expands from ZT08 to ZT0. For comparative analysis, the scotophase was split in the first half of scotophase from ZT08 to ZT16 and in the second half of scotophase from ZT16 to ZT0. Furthermore, the time frame middle of scotophase from ZT12 to ZT20 was used to exclude the potential interference of early torpor onsets and late torpor arousals (Figure 2B). In LP, the photophase expands from ZT0 to ZT16. Accordingly, the scotophase expands from ZT16 to ZT0. The time frames enabled a general description of the absolute locomotor activity extent during the hamsters’ active and resting phases and, furthermore, the calculation of the activity ratios to compare the circadian patterns independent of the individual activity extent. For the photophase-to-scotophase activity ratio, values below 1 indicate a higher activity during scotophase and, therefore, nocturnality. The second-to-first half of scotophase activity ratio was calculated to further characterize the different activity phenotypes, whereby values below 1 indicate a higher activity during the first half of scotophase.

### Statistics and Plotting

Data are given as the mean ± standard deviation of the mean, unless otherwise stated. Statistical tests were performed with SigmaPlot version 11 (Systat Software, San Jose, CA, United States). Data were tested for normality with the Shapiro–Wilk test. One-way repeated measures ANOVA with Holm–Sidak *post hoc* test was used for dependent, normally distributed data; Friedman repeated measures ANOVA with Tukey’s *post hoc* test was used for dependent, non-normally distributed data. A two-tailed *t*-test was used for the comparison of two groups with independent, normally distributed data. Comparisons of more than two groups were done by one-way ANOVA with Holm–Sidak *post hoc* test for independent, normally distributed data and Kruskal–Wallis one-way ANOVA with Dunn’s *post hoc* test for independent, non-normally distributed data. Two dependent datasets per animal were analyzed with the paired *t*-test for normally distributed data and with the Wilcoxon signed-rank test for non-normally distributed data.

Correlation of two parameters is shown in scatterplots. Pearson’s product moment correlation was used to interpret normally distributed parameter correlations, with *p* < 0.05 as statistically significant. Statistically significant correlations are indicated by the *p*-value, the *r*, and the coefficient of determination *R*^2^. Data distribution is shown in boxplots. The middle line represents the median, while the cross stands for the mean value. Each box extends from the 25th percentile to the 75th percentile. The whiskers depict the minimum and maximum values.

In the actograms, consecutive activities per hour were double plotted using the ActogramJ plugin for ImageJ (U.S. National Institutes of Health, Bethesda, MD, United States^1^, 1997–2018). Labeling was done with Inkscape (1.0, ©2020, Inkscape Developers^2^) and Adobe^®^ Photoshop CS2 (9.0, 1990–2005, Adobe Systems Incorporated, San Jose, CA, United States). Activities above the upper limit of 10 cpm are shown as 10 cpm. Since ZT0 was 1 h later in SP, an empty hour was inserted to align SP to LP for the actograms. The day of photoperiod change from LP to SP was excluded as photophase began according to the LP regime and ended according to the SP regime, resulting in an intermediate photophase length of 9 h.

## Results

### Approach 1—Spontaneous Daily Torpor

For this approach, one representative SP week, SP15 ± 2 weeks, was chosen to analyze the adaptation and radiotelemetry parameters of 60 responders in relation to their individual torpor behavior. At least once during their individual data acquisition interval, 46 of the 60 animals expressed torpor, yet not necessarily during the week of analysis, while the remaining 14 animals were never observed in torpor.

In torpor-expressing hamsters (*n* = 46), the torpor incidence during the week of analysis had a median of 0.3 and ranged from 0.0 (no torpor, *n* = 3) to 1.0 (torpor every day, *n* = 1). Below 30°C reached 84 ± 33% of torpor bouts. Additionally, up to four torpor bout attempts were made per individual, with a median incidence of 0.1 for this cohort. The 46 animals showed either a torpor bout attempt or a torpor bout per definition on 55 ± 28% of the analyzed days.

#### Torpor Parameters

Within the week of analysis, the cohort expressed 127 torpor bouts, which were analyzed in detail. Torpor onset was at ZT1.6 ± 1.4 h and torpor offset at ZT5.3 ± 2.0 h (Figure 3). The torpor bouts had a duration of 218 ± 122 min. The minimal body temperature per bout was 26.0 ± 2.8°C. With an earlier torpor onset, a lower minimal body temperature (*r* = 0.385, *p* < 0.05; Figure 3) and a longer torpor bout duration (*r* = -0.384, *p* < 0.05) were reached, despite an earlier torpor offset (*r* = 0.323, *p* < 0.05). Nevertheless, a later torpor offset was generally associated with a longer duration (*r* = 0.750, *p* < 0.05) and a lower minimal body temperature (*r* = -0.628, *p* < 0.05; Figure 3).

To use the torpor parameters for phenotyping, the values were averaged for each of the 42 animals. Consequently, the cohort’s torpor behavior was characterized by a torpor onset at 1.9 ± 1.4 h, a torpor offset at 5.4 ± 1.7 h, a torpor duration of 208 ± 101 min, and a minimal body temperature of 26.3 ± 2.4°C. Hamsters with an earlier mean torpor onset showed a lower standard deviation of their mean torpor onset (hamsters with at least two bouts, *n* = 35, *r* = 0.528, *p* < 0.05) and a higher torpor incidence (*n* = 42, *r* = -0.361, *p* < 0.05).

The majority of torpor bouts had their on- and offsets within the photophase from ZT0 to ZT08 (Figure 3), but six torpor bouts started before ZT0 and ten ended after ZT08. Although some early torpor bouts already started during the second half of the preceding scotophase, and some very long or late torpor bouts reached into the first half of the following scotophase, torpor did not expand into the middle of the scotophase. The lowest body temperatures per hour and hamster recorded during the analysis week were 21.2°C during photophase, 22.9°C during the first half, and 30.7°C during the second half of scotophase, but 35.5°C during middle of scotophase.

#### Torpor Incidence Groups

**FIGURE 4:**
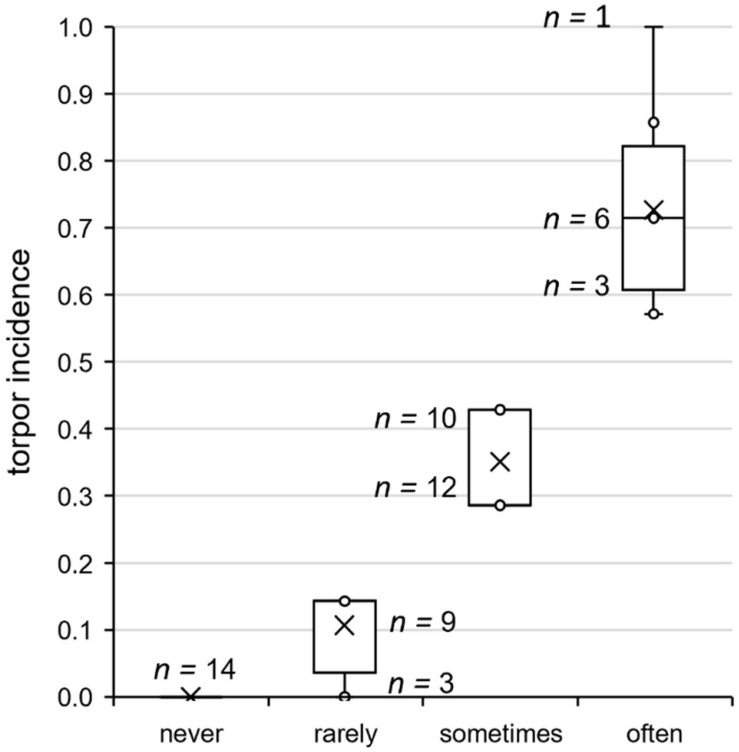
Torpor incidence groups of approach 1. Grouping of all hamsters according to their individual torpor incidence shown in the week of analysis. From the distribution of the torpor incidence values of the hamsters expressing torpor, yet not necessarily during the week of analysis, three groups were derived for phenotyping. Taking the 25th percentile (torpor incidence of 0.2) and the 75th percentile (torpor incidence of 0.5) as thresholds, the animals were assigned to the groups “rarely torpor” (<25%, *n* = 12), “sometimes torpor” (25–75%, *n* = 22), and “often torpor” (>75%, *n* = 12). The torpor incidence varied between hamsters of the same group, as indicated with the sample sizes per torpor incidence value *next to the boxplots*. Responders that never expressed torpor during their individual implantation time accounted for the fourth group “never torpor” (*n* = 14).

After analysis of the torpor parameters, all 60 hamsters were assigned to four torpor incidence groups (Figure 4) to relate torpor behavior, adaptation, body temperature, and activity parameters. Hamsters of the group “never torpor” (*n* = 14) did not express torpor during their entire individual observation interval. Hamsters of the group “rarely torpor” (*n* = 12) were capable of torpor expression, but expressed no or one torpor bout during the week of analysis. Hamsters of the group “sometimes torpor” (*n* = 22) had a torpor incidence between 0.3 and 0.5, while those of the group “often torpor” (*n* = 12) had a torpor incidence of 0.5 or higher. Consequently, the median torpor incidence values were 0.1 in the group “rarely torpor”, 0.3 in the group “sometimes torpor”, and 0.7 in the group “often torpor” (Table 1). The torpor incidence groups differed in torpor onset, with ZT2.6 in hamsters of the group “rarely torpor”, ZT2.0 in hamsters of the group “sometimes torpor”, and ZT1.0 in hamsters of the group “often torpor”.

**TABLE 1:** Phenotyping of the torpor incidence groups according to the torpor parameters of approach 1.

Torpor characteristics	Given values	Torpor incidence group				ANOVA		*Post hoc* test with *p* < 0.050
				
		Never torpor (*n* = 14)	Rarely torpor (*n* = 12)	Sometimes torpor (*n* = 22)	Often torpor (*n* = 12)	Test statistics	*p*-value	Comparison
Torpor per definition incidence	Median	–	**0.1**	**0.3**	**0.7**	*H*_(2)_ = 40.0	<0.001	Rarely *vs*. sometimes
								Rarely *vs*. often
								Sometimes *vs*. often
Torpor attempt incidence	Median	–	**0.0**	**0.2**	**0.1**	*H*_(2)_ = 3.1	0.215	–
Torpor onset (ZT)	Median	–	**2.6**	**2.0**	**1.0**	H_(2)_ = 8.4	0.015	Rarely *vs.* often
								Sometimes *vs.* often
Torpor offset (ZT)	Mean	–	**5.5**	**5.5**	**5.1**	*F*_(41,2)_ = 0.2	0.823	–
Torpor duration (min)	Mean	–	**174**	**200**	**246**	*F*_(41,2)_ = 1.4	0.259	–
Minimal torpor *T*_b_ (°C)	Mean	–	**27.2**	**26.5**	**25.4**	*F*_(41,2)_ = 1.6	0.219	–

*One week was analyzed. The grouping is based on the torpor per definition incidence (Figure 4). Each hamster’s mean value per torpor parameter (onset, offset, duration, and minimal torpor body temperature) was included in the comparison. More details on the statistical tests are listed in Supplementary Table 2.*

**FIGURE 5:**
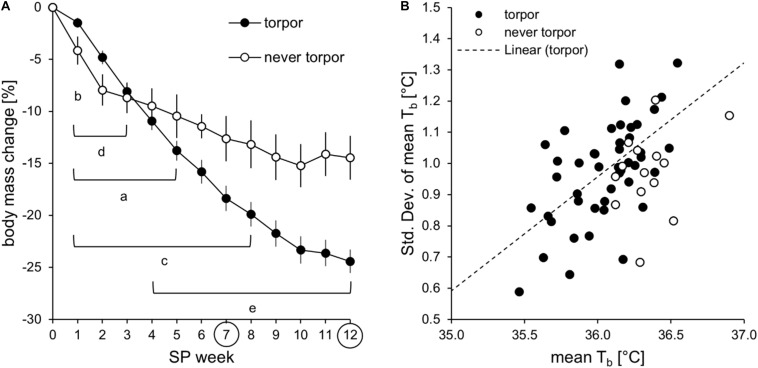
Comparison of hamsters with and without torpor expression of approach 1. **(A)** Mean (±standard error) relative body mass change during adaptation to the short photoperiod [*n*_torpor_ = 46, *n*_never torpor_ = 14]. Significant differences to SP07 of “torpor” (*a*) and “never torpor” (*b*) hamsters. Significant differences to SP12 of “torpor” (*c*) and “never torpor” (*d*) hamsters. Significant differences between “torpor” and “never torpor” hamsters (*e*) [two-way repeated measures ANOVA: *F*_(__11_,_638__)_ = 10.124, *p* < 0.001, with Holm–Sidak *p* < 0.001]. **(B)** Correlation of the mean body temperature and its standard deviation during the middle of scotophase. The higher the mean body temperature, the lower is its standard deviation in “torpor” hamsters (*n* = 46, with *R*^2^ = 0.3489, *r* = 0.591, *p* < 0.050). This correlation could not be confirmed in “never torpor” hamsters.

The adaptation phenotype (Table 2) indicated that the “never torpor” group could be discriminated from the “often torpor” group by absolute body mass in SP12 and from all other torpor incidence groups by the relative body mass change in SP07 and SP12. Regarding no torpor and torpor expression only, “never torpor” hamsters showed a lower relative body mass change than did torpor-expressing hamsters after SP03 (Figure 5A).

**TABLE 2:** Phenotyping of the torpor incidence groups according to the adaptation parameters of approach 1.

Adaptation parameters	Given values	Torpor incidence group				ANOVA		*Post hoc* test with *p* = 0.050
				
		Never torpor (*n* = 14)	Rarely torpor (*n* = 12)	Sometimes torpor (*n* = 22)	Often torpor (*n* = 12)	Test statistics	*p*-value	Comparison
Fur index in SP07	Median	**1.8**	**1.5**	**2.0**	**1.3**	*H*_(3)_ = 3.6	0.310	–
Fur index in SP12	Median	**3.3**	**3.0**	**3.3**	**3.3**	*H*_(3)_ = 0.0	0.998	–
Body mass in SP00 (g)	Mean	**35.0**	**35.9**	**37.2**	**35.3**	*F*_(56,3)_ = 0.6	0.615	–
Body mass in SP07 (g)	Mean	**30.5**	**28.9**	**30.4**	**28.4**	*F*_(56,3)_ = 0.8	0.475	–
Body mass in SP12 (g)	Mean	**29.9**	**27.3**	**28.2**	**25.5**	*F*_(56,3)_ = 3.1	0.035	Never *vs*. often
Body mass change in SP07 (%)	Mean	**–8.7**	**–19.3**	**–17.4**	**–19.2**	*F*_(56,3)_ = 5.0	0.004	Never *vs*. rarely
								Never *vs*. sometimes
								Never *vs*. often
Body mass change in SP12 (%)	Mean	**–10.7**	**–23.5**	**–23.3**	**–27.4**	*F*_(56,3)_ = 12.5	< 0.001	Never vs. rarely
								Never *vs.* sometimes
								Never *vs.* often

*Fur index, scored from 1 for a light brown summer fur to 6 for a dense white winter fur (Figala et al., 1973), and absolute body mass were assessed weekly over the course of adaptation to the short photoperiod. The relative body mass change was calculated in relation to SP00. Three weeks of interest were chosen, namely, SP00: start of adaptation; SP07: before torpor expression; and SP12: after first torpor bouts had been observed. More details on the statistical tests are listed in Supplementary Table 2.*

Activity phenotyping per time frame showed that the “rarely torpor” group was more active than the “often torpor” group during scotophase, first half of scotophase, and middle of scotophase. Body temperature phenotyping per time frame revealed differences between the torpor incidence groups in all time frames (Table 3). Firstly, the “sometimes torpor” and “often torpor” groups had lower body temperatures than the “never torpor” and “rarely torpor” groups, even during the middle of scotophase. Secondly, the “sometimes torpor” and “often torpor” groups could be discriminated by the scotophase body temperature as well as by the standard deviation of the mean body temperature during the middle of scotophase. Thirdly, the groups “never torpor” and “rarely torpor” could be discriminated by the standard deviation of the mean body temperature during the middle of scotophase. The “never torpor” hamsters had higher mean body temperatures within all time frames than all “torpor” hamsters, also during the middle of scotophase [two-tailed *t*-test: *t*_(__58__)_ = -4.026, *p* < 0.001; “torpor”: 36.0 ± 0.0°C SEM; “never torpor”: 36.4 ± 0.1°C SEM)]. The lower the mean body temperature during the middle of scotophase, the smaller was its standard deviation in “torpor” hamsters, while this correlation was not given for the “never torpor” hamsters (Figure 5B).

**TABLE 3:** Phenotyping of the torpor incidence groups of approach 1 according to the activity and body temperature per time frame.

Parameters		Given values	Torpor incidence group				ANOVA		*Post hoc* test with *p* = 0.050
					
			Never torpor (*n* = 14)	Rarely torpor (*n* = 12)	Sometimes torpor (*n* = 22)	Often torpor (*n* = 12)	Test statistics	*p*-value	Comparison
Activity	Total (cpm)	Median	**4.0**	**4.8**	**4.4**	**2.9**	*H*_(3)_ = 7.4	0.060	–
	Photophase (cpm)	Median	**2.1**	**2.0**	**2.0**	**1.5**	H_(3)_ = 3.4	0.334	–
	Scotophase (cpm)	Median	**5.1**	**6.0**	**5.5**	**3.6**	*H*_(3)_ = 9.2	0.026	Rarely *vs*. often
	First half of scotophase (cpm)	Median	**5.8**	**8.0**	**6.9**	**3.9**	*H*_(3)_ = 10.2	0.017	Rarely *vs.* often
	Second. half of scotophase (cpm)	Median	**4.4**	**4.3**	**4.0**	**3.4**	*H*_(3)_ = 4.1	0.248	–
	Middle of scotophase (cpm)	Median	**4.9**	**6.0**	**5.5**	**3.7**	*H*_(3)_ = 8.4	0.039	Rarely *vs*. often
	Std. Dev. of middle of scotophase (cpm)	Median	**10.3**	**13.0**	**11.7**	**9.5**	*H*_(3)_ = 4.8	0.189	–
	Photophase-to-scotophase ratio	Median	**0.4**	**0.4**	**0.4**	**0.5**	*H*_(3)_ = 0.3	0.951	–
	Second-to-first half of scotophase ratio	Median	**0.7**	**0.5**	**0.7**	**0.8**	*H*_(3)_ = 6.8	0.078	–
Body temperature	Total (°C)	Median	**36.1**	**35.8**	**35.4**	**34.6**	*H*_(3)_ = 47.8	<0.001	Never *vs*. sometimes
									Never *vs*. often
									Rarely *vs*. sometimes
									Rarely *vs*. often
	Photophase (°C)	Median	**35.7**	**35.1**	**34.3**	**32.1**	*H*_(3)_ = 47.0	< 0.001	Never *vs.* sometimes
									Never *vs.* often
									Rarely *vs.* sometimes
									Rarely *vs.* often
	Scotophase (°C)	Mean	**36.4**	**36.2**	**36.0**	**35.8**	*F*_(56,3)_ = 18.5	<0.001	Never *vs*. sometimes
									Never *vs*. often
									Rarely *vs*. sometimes
									Rarely *vs*. often
									Sometimes *vs*. often
	First half of scotophase (°C)	Median	**36.5**	**36.4**	**36.3**	**35.9**	*H*_(3)_ = 27.7	<0.001	Never *vs*. sometimes
									Never *vs*. often
									Rarely *vs*. often
	Second half of scotophase (°C)	Median	**36.2**	**36.0**	**35.9**	**35.6**	*H*_(3)_ = 27.5	<0.001	Never *vs*. sometimes
									Never *vs*. often
	Middle of scotophase (°C)	Mean	**36.3**	**36.3**	**36.0**	**35.9**	*F*_(56,3)_ = 13.4	<0.001	Never *vs*. sometimes
									Never *vs*. often
									Rarely *vs*. sometimes
									Rarely *vs*. often
	Standard deviation of middle of scotophase (°C)	Mean	**0.97**	**1.1**	**0.98**	**0.84**	*F*_(56,3)_ = 7.8	<0.001	Never *vs*. rarely
									Never *vs*. often
									Rarely *vs*. often
									Sometimes *vs*. often

*Data of one analysis week were used. More details on the statistical test results are listed in Supplementary Table 3. The lower the photophase-to-scotophase activity ratio, the higher the degree of nocturnality. The lower the second-to-first half of scotophase activity ratio, the higher the activity during the first half of scotophase.*

### Approach 2—Initial and Long-Term Effects of Short Photoperiod

Twenty-three percent of the hamsters which adapted to SP between 2018 and 2020 never expressed torpor. In approach 1, they could be discriminated from the torpor-expressing hamsters by the weaker body mass reduction and, thus, already during SP adaptation. Since 11 of the 60 hamsters had already been implanted before the beginning of SP, their body temperature and activity profiles were analyzed during the change from LP to SP and the following SP adaptation to identify potential additional predictors of torpor proneness early during adaptation. Due to the low sample size, comparisons of “torpor” hamsters (*n* = 8) and “never torpor” hamsters (*n* = 3) were statistically invalid. The values per parameter, time frame, experimental week, and individual are given in the supplementary tables (Supplementary Tables 4–8).

#### Body Temperature

**FIGURE 6:**
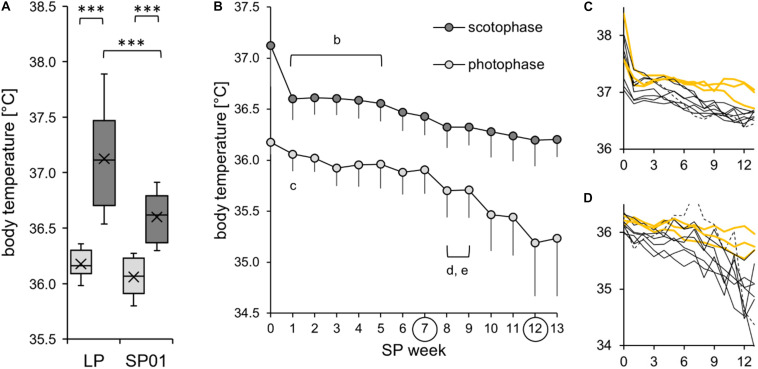
Body temperature during the short photoperiod adaptation of approach 2. The exact values per hamster can be found in Supplementary Table 4. **(A)** The cohort’s initial reduction of body temperature after the change of the light regime (*n* = 10). Comparison of body temperature during photophase (*light gray*) and scotophase (*dark gray*) of the last week of the long photoperiod (*LP*) and the first week of the short photoperiod (*SP01*). According to a two-way repeated measures ANOVA, body temperature was affected by both time frame [*F*_(__1_,_9__)_ = 62.256, *p* < 0.001, power = 1.0] and light regime [*F*_(__1_,_9__)_ = 83.125, *p* < 0.001, power = 1.0]. In addition, there was an interaction between the effects of time frame and light regime [*F*_(__1_,_9__)_ = 15.373, *p* = 0.004, power = 0.9). *Post hoc* Holm–Sidak tests confirmed that body temperature was significantly higher during the scotophase in both LP and SP01 (***p < 0.001). Furthermore, body temperature during the scotophase (***p < 0.001) was significantly higher in LP than that in SP01. Hamster #06 was excluded because it performed a torpor bout on the second day of SP, which is atypical or usually not noticed. **(B)** The cohort’s further reduction of body temperature (*n* = 10). With focus on the weeks from SP01 to SP07 without torpor expression, body temperature was reduced over the course of SP adaptation. Significant differences to the SP07 of scotophase (*b*) [one-way repeated measures ANOVA: *F*_(__6_,_54__)_ = 7.599, *p* < 0.001] as well as of photophase (*c*) [one-way repeated measures ANOVA: *F*_(__6_,_54__)_ = 4.851, *p* < 0.001], both confirmed with Holm–Sidak *p* < 0.001. With focus on the weeks from SP08 to SP12 including torpor bouts, body temperature was further decreased. Significant differences to the SP12 for scotophase (*d*) [one-way repeated measures ANOVA: *F*_(__5_,_45__)_ = 4.978, *p* = 0.001, with Holm–Sidak *p* = 0.002] as well as for photophase (*e*) [Friedman repeated measures ANOVA: *χ*^2^_(__5__)_ = 30.114, *p* < 0.001, with Tukey’s test *p* < 0.05]. Hyperactive hamster #06 was excluded since a stereotypic jumping during photophase was also reflected in the body temperature. **(C,D)** Individual reduction of body temperature during scotophase **(C)** and photophase **(D)** for each of the 11 hamsters. Body temperature (in degree Celsius) is shown on the *y*-axis, the SP week on the *x*-axis, with hamster #06 as *dotted line*, the three “never torpor” hamsters as *yellow lines*, and all others as *thin black lines*.

To assess the initial effects of SP exposure, the cohort’s body temperature measurements of the last week in LP and the first week in SP were compared (Figure 6A). In both LP and SP01, body temperature was significantly lower during the photophase than during the scotophase, while the reduction from LP to SP01 was only significant during the scotophase. The body temperature of the cohort further decreased over the course of SP adaptation (Figure 6B). The individual development of body temperature during the scoto- and photophases was diverse (Figures 6C,D). Hamster #06 was excluded from this and most other cohort models, as indicated in the figure legends. It expressed a torpor bout on the second day of SP, which is atypical or usually not noticed. It further developed a stereotypic jumping during photophase from SP04 to SP11.

First torpor bouts were expressed in SP07 (first torpor of hamster #03), SP08 (hamster #11), SP09 (hamsters #06 and #08), SP10 (hamsters #05 and #07), and SP11 (hamsters #09 and #10), with a median of 67 days of SP adaptation. The eight hamsters expressed between one and 12 torpor bouts until SP13, with a median of four. The absolute number of torpor bouts from SP08 to SP13 was positively correlated with the torpor incidence of SP13 (*n* = 8, *r* = 0.948, *p* < 0.05), the week of torpor analysis in approach 1. Three hamsters (#01, #02, and #12) did not express torpor until termination in SP14.

**FIGURE 7:**
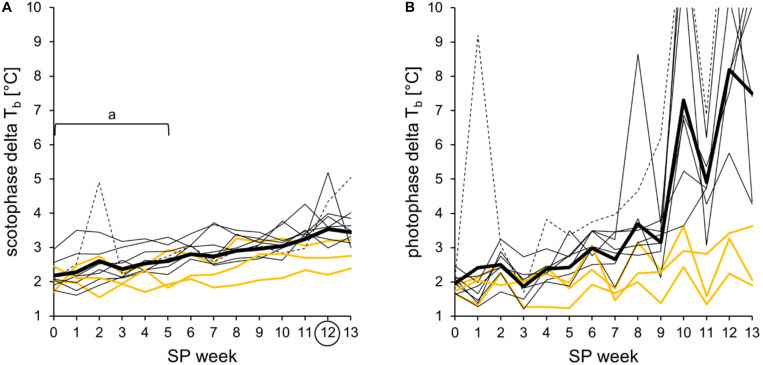
Delta body temperature during the short photoperiod adaptation of approach 2. The difference between the highest value per hour and the lowest value per hour, detected within a time frame, is shown for each animal and week. Hamster #06 is represented as *dotted line*, the three “never torpor” hamsters as *yellow lines*, all others as *thin black lines*, and the cohort’s mean delta body temperature as *thick black line*. The exact values per hamster can be found in Supplementary Table 5. **(A)** Delta body temperature of scotophase. The values per individual were averaged as delta of the cohort (*n* = 11, *thick line*), which showed a linear development (from SP00 to SP13: *R*^2^ = 0.936, *r* = 0.967, *p* < 0.05). For the cohort, significant differences to SP12 are indicated with “*a*” [Friedman repeated measures ANOVA: *χ*^2^_(__13__)_ = 93.566, *p* < 0.001, with Tukey’s test *p* < 0.05]. **(B)** Delta body temperature of photophase. Spontaneous daily torpor, irregularly expressed by hamster #06 in SP01 and regularly expressed by eight of the 11 hamsters after SP07, caused high delta body temperatures during photophase.

In this study, the delta body temperature was used as an indicator of the body temperature spectrum covered by the hamsters. It was calculated by subtracting the minimal body temperature per hour from the maximal body temperature per hour individually detected within a time frame per week. The cohort’s scotophase delta was significantly lower in SP00 until SP05 when compared to SP12. Furthermore, it increased linearly in all 11 hamsters over the course of SP adaptation (Figure 7A). In contrast, the photophase delta was highly individual, especially when including torpor expression after SP07. Until SP13, the photophase delta rose to maximal 14.4°C, reflecting the difference between the coldest and the warmest hour of an animal within photophase and week (Figure 7B).

#### Activity

**FIGURE 8:**
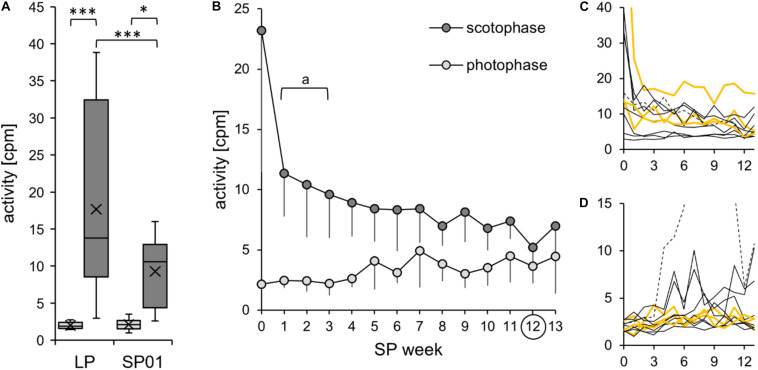
Activity during the short photoperiod adaptation of approach 2. The exact values per hamster can be found in Supplementary Table 6. **(A)** The cohort’s initial adaptation of activity after the change of the light regime (*n* = 10). Comparison of activity during photophase (*light gray*) and scotophase (*dark gray*) of the last week of the long photoperiod (*LP*) and the first week of the short photoperiod (*SP01*). According to the two-way repeated measures ANOVA, the activity was affected by both time frame [*F*_(__1_,_9__)_ = 21.155, *p* = 0.001, power = 0.984] and light regime [*F*_(__1_,_9__)_ = 7.689, *p* = 0.022, power = 0.637]. In addition, there was an interaction between the effects of time frame and light regime [*F*_(__1_,_9__)_ = 7.815, *p* = 0.021, power = 0.645]. *Post hoc* Holm–Sidak tests confirmed that the activity was significantly higher in LP scotophase (***p < 0.001) and in SP01 (*p < 0.026) compared to that in photophase. Furthermore, scotophase activity was higher in LP than that in SP01 (***p < 0.001), yet with a low power. Hamster #02 was excluded from the cohort’s model since it had an outlying scotophase activity level in LP (beyond the three times interquartile distance threshold of the cohort), with a drastic initial decrease from 79 cpm during LP scotophase to 25 cpm during SP01 scotophase. **(B)** Further activity development of the cohort (*n* = 6). When comparing the adaptation period from SP01 to SP12, the scotophase activity was reduced. Significant differences to SP12 are indicated with “*a*” [one-way repeated measures ANOVA: *F*_(__12_,_60__)_ = 4.750, *p* < 0.001, with Holm–Sidak *p* < 0.001]. The photophase activity slightly rose, yet with a significant difference from SP04 to SP08 only [Friedman repeated measures ANOVA: *χ*^2^_(__13__)_ = 36.229, *p* < 0.001, with Tukey’s test *p* < 0.05]. Excluded were hamster #01, with high LP activity and an extremely slow activity adaptation to SP; hamster #02, with outlying LP activity; hamster #06, with stereotypic jumping behavior in the cage corners during photophase from SP04 to SP11; and the hamsters #10 and #11, which were extremely calm and did not modulate initial activity. **(C,D)** Individual reduction of body temperature during scotophase **(C)** and photophase **(D)** for each of the 11 hamsters. Activity (in counts per minute) is displayed on the *y*-axis, the SP week on the *x*-axis with hamster #06 as *dotted line*, the three “never torpor” hamsters as *yellow lines*, and all others as *thin black lines*.

To assess the initial effects of SP exposure, the cohort’s activity measurements of the last week in LP and the first week in SP were compared (Figure 8A). In the first week of SP, the cohort reduced its activity during scotophase, while its activity during the photophase remained constant. In both photoperiods, the hamsters were nocturnal, with a higher activity during scotophase.

According to observations and the radiotelemetry data, the activity levels strongly differed due to the hamsters’ broad behavioral spectrum from calm to active. The highest activity per hour determined in each hamster’s LP scotophase varied from 11 cpm in the calmest to 158 cpm in the most active hamster, while the variations during the LP photophase ranged from 6 to 60 cpm. In contrast, the highest activity per hour in SP01 varied from 12 to 101 cpm during the scotophase and from 5 to 26 cpm during the photophase.

**FIGURE 9:**
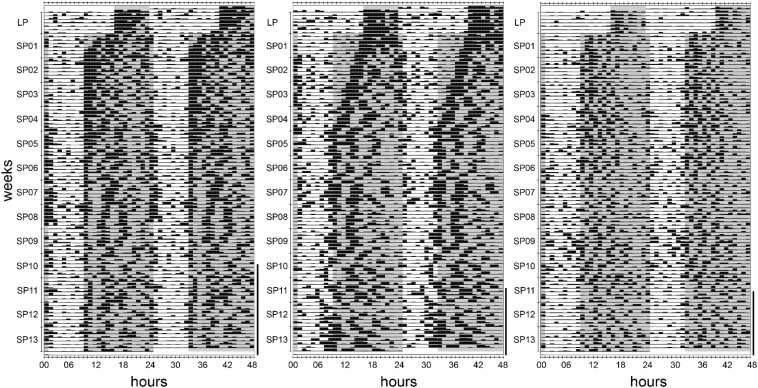
Actograms of three individuals of approach 2 during 1 week in the long photoperiod and adaptation to the short photoperiod. *Black bars* indicate the activity performed within an hour, while values from 0 to 10 cpm are displayed. The long photoperiod (LP) and short photoperiod (SP) scotophases are indicated with *gray areas*. The days following the first torpor bout are marked with a *vertical line on the right side* of each actogram. All 11 hamsters adapted gradually to the immediate change from LP to SP, however at different paces. The *left actogram* (hamster #07) serves as an example for the majority of hamsters that showed a fast adaptation within days. The *middle actogram* (hamster #09) represents one of two hamsters with a gradual adaptation within weeks. The *right actogram* represents one of two hamsters showing a fast adaptation, but a very low activity level that impeded the analysis of temporal activity organization (hamster #10).

Over the course of 13 weeks in SP, the scotophase activity was further reduced in general, while the photophase activity slightly increased (Figure 8B). However, the scoto- and photophase activities were differently modulated by the 11 hamsters (Figures 8C,D). Activity onset occurred sharply at the beginning of the scotophase, while activity faded over the course of the scotophase so that the offset was difficult to define (Figure 9). The hamsters adapted to the immediate change from LP to SP by expanding their activity gradually into the prolonged scotophase and shifted their activity peak to the new beginning of the scotophase. According to eye fitting using actograms, seven animals adapted their activity within the first SP week and two others during the second SP week, while adaptation occurred after 4 weeks in hamster #09 and after 8 weeks in the “never torpor” hamster #01.

**FIGURE 10:**
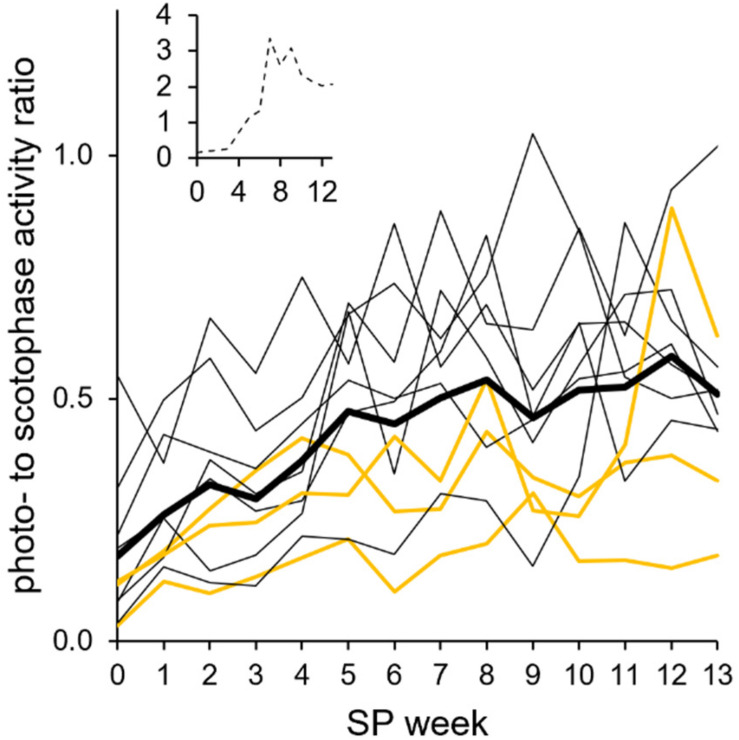
Photophase-to-scotophase activity ratio during the short photoperiod adaptation of approach 2. For each animal and week, the photophase-to-scotophase activity ratio was calculated to identify nocturnality (ratio < 1) and diurnality (ratio > 1). Individual ratios (the three “never torpor” hamsters as *yellow lines* and all others as *thin black lines*; *inset*: graph of hyperactive hamster #06) were averaged for the cohort’s ratio (*thick line*). The cohort’s ratio (*n* = 10, without hamster #06) showed a positive linear correlation with progressing adaptation to the short photoperiod (SP) (*R*^2^ = 0.8144, *r* = 0.902, *p* < 0.05). Significant differences to SP07 before torpor expression were found in SP00 and SP01, while differences to SP12 occurred in SP00, SP01, and SP03 [Friedman repeated measures ANOVA: *χ*^2^_(__13__)_ = 68.811, *p* < 0.001, with Tukey’s test *p* < 0.05]. The exact values per hamster can be found in Supplementary Table 7.

The hamsters’ nocturnality was additionally confirmed by a photophase-to-scotophase activity ratio calculated per hamster and week (Figure 10). Ratios below 1 indicate a higher activity during scotophase and, therefore, nocturnality. Nocturnality of the cohort was most pronounced in LP and became weaker until SP05 (the ratio increased from about 0.2 to 0.5). From SP05, the ratio remained at about 0.5, indicating that the photophase activity was half that of the scotophase activity. The hyperactivity hamster #06 expressed during photophase after SP04 was reflected by ratios higher than 1, indicating diurnality (Figure 10, insert).

The cohort maintained a higher activity in the first than in the second half of scotophase, with an average second-to-first half of scotophase activity ratio smaller than 1, namely, 0.7 ± 0.3 in SP01 and 0.6 ± 0.2 in later weeks. Exceptions were a ratio of 1.7 in hamster #09 during SP01 and a ratio of 1.3 in hamster #07 during SP09, as they were more active in the second half of the scotophase. The extremely slow activity adaptation of hamster #01 was reflected by a ratio decline from 8.3 in SP01 to 0.7 in SP13, with ratios smaller than 1 after SP08.

#### Body Mass and Fur

**FIGURE 11:**
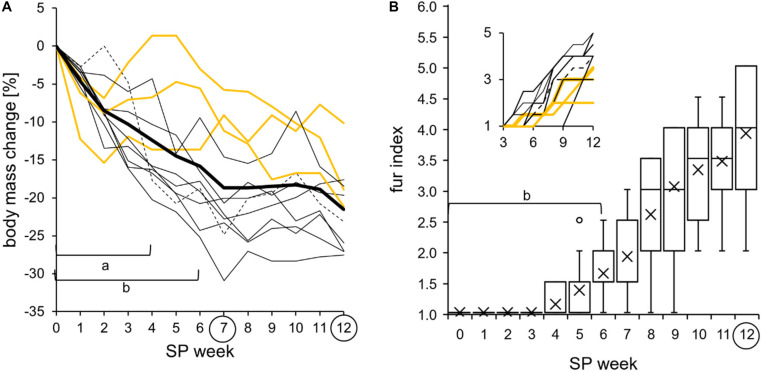
Body mass change and molt into a white winter fur during the short photoperiod adaptation of approach 2 (*n* = 11). The exact values per hamster can be found in Supplementary Table 8. **(A)** Relative body mass change with the long photoperiod (LP) as baseline. The cohort’s significant differences of the relative body mass change (*thick line*) to SP07 are indicated with “*a*” e.g., to SP12 with “*b*” [one-way repeated measures ANOVA: *F*_(__12_,_120__)_ = 28.777, *p* < 0.001, with Holm–Sidak *p* < 0.001]. All hamsters changed their body mass during the short photoperiod (SP) adaptation (hamster #06 as *dotted line*, the three “never torpor” hamsters as *yellow lines*, and all others as *thin black lines*). **(B)** Fur index from 1 for summer fur to 6 for winter fur. The cohort’s significant differences of fur index (*boxplots*) to SP12 are indicated with “*b*” [Friedman repeated measures ANOVA: *χ*^2^_(__12__)_ = 122.375, *p* < 0.001, with Tukey’s test *p* < 0.05]. All hamsters increased their fur index. *Inset*: graph with color code of graph **(A)**.

All hamsters reduced their body mass during SP adaptation (Figure 11A). The cohort had an initial body mass of 32.7 ± 5.2 g, which was reduced by -19 ± 8% to 26.5 ± 4.3 g in SP07. “Never torpor” hamsters seem to have a less drastic reduction of body mass, as already shown in approach 1 (Figure 5A). All hamsters changed their light brown summer fur to a dense white winter fur over the course of SP adaptation (Figure 11B). The first changes became visible in SP04 in three of 11 animals. Hamster #08 started to change its fur in SP10, when most other animals already finished their fur change.

## Discussion

In the present study, the high variability of the adaptation parameters and torpor use in Djungarian hamsters was confirmed for all the observed parameters; however, the two analytic approaches for a large and detailed sample set also revealed new aspects. Overall, the hamster colony at Ulm University resembles the animals in early reports after domestication (Figala et al., 1973; Ross, 1998), yet with a weaker adaptation response. This likely results from the high ambient temperature of 20°C, corresponding to the lower limit of the hamsters’ thermoneutral zone when SP-adapted (Heldmaier and Steinlechner, 1981a). Although the present animals have a domestication history of 50 years, outbreeding could preserve their circadian and seasonal phenotype, i.e., nocturnality as well as a body mass reduction of 20%, a fur index between 3 and 5, and torpor expression in most animals.

### Torpor

The expression of spontaneous daily torpor in Djungarian hamsters has been shown to be under strict circadian control (Ruf et al., 1989; Ruby and Zucker, 1992). The analyses of the present study, however, indicate a rather flexible timing of torpor during photophase (Figure 3 and Table 1). The 127 torpor bouts analyzed had their onset at ZT1.6 ± 1.4 h. It is important to notice that the reduction of the metabolic rate below the resting metabolic rate during torpor entrance precedes the reduction of body temperature below 32°C by almost an hour (Ruf and Heldmaier, 2000; Heldmaier et al., 2004). Consequently, all torpor onsets determined by body temperature measurements before ZT01 already occurred before the end of the scotophase and thus without light as a proximate induction stimulus.

The 46 torpor-expressing hamsters analyzed in approach 1 had shown at least one torpor bout within their individual observation interval. For reasons of comparability, the approach analyzed one representative SP week during the torpor period, resulting in a median torpor incidence of 0.3 with some hamsters that did not show torpor in this specific week. The respective torpor parameters were analyzed for single torpor bouts irrespective of animal (*n* = 127) and means per animal (*n* = 42). Both analyses revealed useful correlations for future interpretations of individual torpor behavior. Increasing the torpor incidence resulted in a higher probability of early and therefore deeper and longer bouts with an early torpor offset, and *vice versa*. Interestingly, torpor onset and its standard deviation correlated positively in the cohort, indicating a lower variability of early torpor bouts and an increasing degree of synchronization to ZT0 with increasing torpor incidence.

Torpor bouts that start early may favor energy saving because they resulted in lower minimal body temperatures per bout and longer torpor bouts in the present study (see also Ruf and Heldmaier, 1992). The hypothermic body temperature and the reduced activity at the beginning of the scotophase additionally indicate post-torpor effects on the hamsters’ scotophase behavior (Table 3). Earlier studies have shown that the hamsters’ circadian rhythm diminishes in SP, whereby torpor might impair sleep during the resting phase (Deboer et al., 2000; Deboer and Tobler, 2003; Scherbarth and Steinlechner, 2008). Time might get lost for feeding, and this energy deficit might directly demand for the next even deeper and longer torpor bouts. This is supported by the observations of a low scotophase activity coinciding with a low scotophase body temperature (Table 3), consequently a smaller standard deviation of body temperature (Figure 5B), and a higher torpor incidence with early, deep, and long torpor bouts (Table 1 and Figure 3).

Furthermore, the present study revealed a remarkable incidence of torpor bout attempts in torpor-expressing hamsters (Table 1) never reported in the literature. They started at the same time and in the same shape as torpor bouts, but were interrupted at about 33°C (Figure 1). It is unclear whether torpor bouts were interrupted by yet undefined external disturbances or due to internal physiological factors. So far, the energy-saving potential of torpor bout attempts remains unknown, but should be examined *via* metabolic rate measurements in conditions carefully controlled for external disturbance factors as they might also reveal new insights into the other functions of torpor (Geiser and Brigham, 2012).

Rare reports of torpor expression without SP adaptation or food restriction exist (Steinlechner et al., 1986). Hamster #06 expressed an irregular torpor bout on the second SP day with an onset at ZT23, which accounts for the ZT0 of LP. Like the other hamsters of the cohort, hamster #06 started to express torpor bouts regularly in SP09 and had a torpor incidence of 0.7 (“often torpor”) in SP13.

The torpor analysis of this study is not free of bias. Data analysis started and ended at ZT0 (beginning of photophase). While individual torpor incidence included all recorded torpor bouts per hamster, torpor bouts starting before ZT0 on the first day of the analysis week could not be analyzed in detail. An alternative data analysis starting and ending at ZT08 (beginning of scotophase) would have resulted in a much higher number of incomplete torpor bouts, as torpor offset after ZT08 was more common than torpor onset before ZT0. Furthermore, the present results show that torpor affected the second half of scotophase prior to torpor as well as the first half of scotophase after torpor. Consequently, the choice of the start and end of an observation interval must be considered thoroughly regarding aims of future studies.

### Sampling Paradigm

The phenotyping of the torpor incidence groups was also used to reassess previous organ sampling schemes (Herwig et al., 2007; Bank et al., 2017; Cubuk et al., 2017a, b). In these sampling paradigms, hypothermic (HT) hamsters were sampled at torpor onset (ZT01), deep torpor (ZT04), torpor offset (ZT07), and after torpor (ZT16), along with time-matched normothermic (NT) hamsters. As there are high inter-individual variabilities of the torpor incidence, onset, depth, and duration within a cohort, not every hamster can be sampled for each group, which impedes a random assignment beforehand. Assuming that the individual torpor timing and torpor incidence are an integrative part of the hamster’s long-term adaptational response, which is determined by a set of hitherto unknown genetic and environmental factors, the data might reflect not only acute effects but also prerequisites of torpor behavior. Consequently, the individual torpor behavior must be determined beforehand to achieve an equal distribution among sampling groups.

The majority of torpor-expressing hamsters (22 out of 46, 48%) showed torpor sometimes (incidence between 0.3 and 0.5; Figure 4) and fit best for all the sampling groups and time points (Table 1). However, torpor attempt incidence is highest in these hamsters (Table 2). A torpor bout attempt would not allow sampling for the particular day since the hamster was neither in torpor per definition nor constantly normothermic (Figure 1A). Hamsters never or rarely expressing torpor are indeed likely to be sampled as normothermic controls. Yet, given their flexible torpor timing, they are also adequate for sampling in hypothermia at ZT01 and ZT07. Hamsters with a high torpor incidence are more likely to be sampled for the hypothermic sampling group, but are not always adequate for sampling at ZT01 and ZT07 since they tended to be almost in deep torpor at ZT01 and finished the torpor bout before ZT07 (Figure 3). Thus, it cannot be assumed that hamsters often expressing torpor are all sampled for the HT group and those that express torpor rarely are all sampled in the NT group.

In future studies, the torpor behavior should be assessed in detail during 1 week of radiotelemetry tracking before sampling. Respective conclusions can be drawn to plan the assignment of each hamster to a certain sampling group in the following week since a stable torpor behavior from one to the next week was assumed due to the positive correlation between the absolute number of torpor bouts until SP12 and the torpor incidence in SP13 of approach 2.

However, this study also indicates restrictions of the rigid yet adequate sampling paradigm. The described flexibility in torpor incidence, timing of torpor, and course of body temperature during a torpor bout is respected in the paradigm as well as possible. An expansion of the sampling time points, from, e.g., ZT01 to ZT0–ZT02, would be eligible for studies on hypo- and normothermia alone, but inconvenient for studies on the circadian rhythm of hypo- and normothermic hamsters. Adapting the 32°C torpor definition threshold is impeded by both torpor bout attempts, which might be misinterpreted as torpor bouts, and the high minimal body temperatures of hamsters rarely expressing torpor. In relation to this, it should be mentioned that the torpor definition threshold of the present and many earlier studies (32°C for at least 30 min) is under constant debate (Boyles et al., 2011; Brigham et al., 2011) and might be refined by including metabolic rate measurements (Diedrich et al., 2015).

### Never Torpor

The present study enables a better identification of responders without torpor expression. In approach 1, 14 “never torpor” hamsters out of a cohort with 60 individuals responded more weakly to SP in terms of body mass reduction (Figure 5A and Table 2). A high mean body temperature with a low standard deviation during the middle of scotophase allows discriminating “never torpor” hamsters not only from “torpor” hamsters but also from “rarely torpor” hamsters, with no to one torpor bout within the analysis week (Figure 5B and Table 3). Yet, due to the small sample size of the early implanted hamsters of approach 2 with three “never torpor” hamsters out of 11, this study cannot reveal at which time point of adaptation these differences became significant. However, an identification of the “never torpor” hamsters 2 weeks after implantation is not sufficient to reduce the number of animals in an experiment or the number of invasive transmitter implantations.

In approach 2, “never torpor” hamsters appeared to respond more weakly in all the observed parameters, suggesting a different metabolic programming of hamsters never expressing torpor at given circumstances (Cubuk et al., 2016; Diedrich et al., 2020). The most promising indicators of “never torpor” hamsters might be a higher body temperature during scotophase over the entire course of SP adaptation (Figure 6C) and a smaller delta body temperature and, therefore, variation during both scoto- and photophases (Figure 7). Indirect calorimetry could be used as a non-invasive alternative to extrapolate from individual fluctuations of metabolic rate to those of body temperature and, thus, torpor or no torpor expression. Body temperature and the metabolic rate correlate (Heldmaier and Ruf, 1992), and our own preliminary measurements in SP-adapted hamsters regarding the middle of scotophase also revealed a positive correlation between the standard deviation of the mean body temperature and the standard deviation of the mean metabolic rate (*n* = 7, *R*^2^ = 0.74, *r* = 0.86, *p* = 0.006). Furthermore, non-invasive infrared cameras with tracking software would enable more attention on the peculiarities regarding the activity levels and activity patterns of “never torpor” hamsters, e.g., a high scotophase activity in LP (hamster #02), a very slow activity adaptation to SP (hamster #01), as well as a photophase-to-scotophase activity ratio below the cohort’s mean, suggesting a more pronounced nocturnality in “never torpor” hamsters (Figure 10).

### Next Torpor

Besides the early identification of torpor-expressing responders, the acute prediction of the next torpor bout in individual hamsters would be of interest to improve planning of sampling. While a study on marsupial sugar gliders has suggested reductions of activity and body temperature as acute predictors of torpor (Christian and Geiser, 2007), efforts to use radiotelemetry data have been hitherto without success. A relation between the activity pattern and the torpor behavior was not found (Figures 1, 2, 9), but the activity ratios on, e.g., a daily instead of a weekly basis, a refined correlation analysis between activity and body temperature, and the standard deviations of the mean values seem to be promising (Figures 5, 10, 11). The most reliable parameter for acute torpor prediction has been and still is the previous torpor incidence, which seems to be rather stable within an individual.

### Adaptation to Short Photoperiod

Djungarian hamsters should be more active in LP than in SP since they expect mating and the intense care for their litters. The resulting high energy demand requires a high foraging activity, which can be reduced during the early stages of SP adaptation as a function of decreasing food intake, body mass, and reproductive activity (Ruf and Heldmaier, 2000). Indeed, a high activity and body temperature in LP and lower values when SP-adapted have been reported (Hamann, 1987; Prendergast et al., 2013) and were confirmed in this study. While body temperature was lower in SP during all time frames (Figure 6), activity was similar in both LP and SP photophase and differed only in scotophase (Figure 8).

The transition of the activity and body temperature patterns from LP to SP has not yet been described for hamsters kept in artificial photoperiods. In this study, the hamsters were subjected to an abrupt light shift from LP to SP, whereby the photophase began 1 h later and ended 7 h earlier. This manner of transition has been used for several hamster cohorts and experiments at Ulm University to allow for the described sampling paradigm when SP-adapted. Consequently, the responses shown in this study may have reflected this specific circadian phase entrainment to the photic *zeitgeber*. Regardless of the transition pattern, the potential influence of a certain shift on the hamster’s individual adaptation and torpor capability must be considered. A fast activity adaptation was previously shown for diverse complex light regime changes, yet with high variations within the cohort including individuals with a low or no adaptation performance (Gorman and Elliott, 2004). Most hamsters were able to re-entrain to a ZT0 shift by 5 h (16:8 h) within 14 days, while some showed a free-running activity pattern or even arrhythmia (Barakat et al., 2004, 2005). In the present study, the majority of hamsters showed a fast response to the new light regime by an immediate synchronization as well as reductions of activity and body temperature during the first SP week (Figures 6A, 8A). In contrast, the body mass and fur index, two parameters strongly influencing thermoregulation, remained initially unchanged (Figure 11).

Activity requires an increase in the metabolic rate, which produces heat and increases body temperature. Thus, the immediate reduction of the scotophase body temperature after the change from LP to SP was largely attributable to the immediate decrease in scotophase activity, although the velocity of this change was rather impressive. As activity recordings have been shown to largely reflect feeding bouts (Ruf and Heldmaier, 1993), the activity decrease observed in this study might have resulted from a decreased drive to feed in order to initiate body mass reduction during SP adaptation (Knopper and Boily, 2000). Earlier studies have already measured lower scoto- and photophase body temperatures in SP- than in LP-adapted Djungarian hamsters (Heldmaier and Steinlechner, 1981a; Korhonen et al., 2008). The present study, however, showed for the first time that this decrease in body temperature already occurs at the very beginning of the SP adaptation and further proceeds during the adaptation process (Figure 6). The hamsters appeared to tolerate the rather acute body temperature reduction and additionally showed an increasing difference between the maximum and minimum body temperatures prior to the beginning of torpor expression, surprisingly not only during photophase but also during scotophase (Figure 7). This observation might be a first indication for an SP-induced early change in body temperature set point, which is gradually adjusted and integrated in the complex morphological and physiological adaptation processes until the beginning of the torpor period. Although this study cannot provide sufficient information on the mechanisms behind the body temperature adjustments, it can be assumed that they contribute to the overall energy-saving purpose of the Djungarian hamster’s adaptative syndrome (Heldmaier and Lynch, 1986).

The high variability in adaptation is considered to be natural and not indicative of the negative effects of domestication and genetic bottlenecks (Figala et al., 1973), as a hamster population should benefit from a certain degree of individual flexibility and variation to cope with acute and unpredictable changes in environmental conditions (Ruf et al., 1991, 1993). Nevertheless, since the Siberian winter is usually long and harsh, nature should have selected for hamsters with a fast and strong adaptation in response to the decreasing photoperiod length, followed by flexible torpor use as anticipation of acute energetic challenges, which are not given in laboratory conditions (Diedrich et al., 2015, 2020).

### Nocturnality

According to the developer, the DSI activity data indicate no, low, or high activity per 3-min recording interval. The method developed for this study made the activity data accessible in more detail by using larger data bins, namely, time frames per week, which leveled natural and technical outliers, while a sufficient time increment was maintained for both long- and short-term observations (Figures 1, 2). Nevertheless, the absolute activity data, recorded and analyzed in cpm, are based on the signal strength changes, which might slightly vary with position and speed the transmitter moving over its receiver. The activity mean values of several animals must be interpreted with caution (Table 3 and Figure 8), while the introduced activity ratios are a promising tool to characterize activity in relative terms with each animal as its own control (Table 3 and Figure 10).

For the present study, using both absolute and relative activity data complemented and verified each other. A higher activity during scotophase than during photophase has been described for rodents in general and has incidentally been shown for Djungarian hamsters (Wynne-Edwards et al., 1999; Refinetti, 2006; Weinert et al., 2009), which could be confirmed in the present study (Figures 8–10, Table 3, and Supplementary Table 6). Previously, different circadian phenotypes have been described, ranging from wild type over delayed onset and arrhythmic to non-responder (Margraf et al., 1991; Gorman and Zucker, 1997; Schöttner et al., 2011). In the present study, all hamsters were nocturnal, with higher absolute activity values during the scotophase and a photophase-to-scotophase activity ratio below 1, while hamster #06’s hyperactivity during the photophase was reflected by photophase-to-scotophase activity ratios higher than 1, proving diurnality. Unlike the other hamsters of the cohort, the calm sibling hamsters #10 and #11, did not decrease their activity over the course of SP adaptation, probably because they could not fall below the basal activity level. Their nocturnality was difficult to detect due to the irregular and shallow activity bouts, but was proven with the photophase-to-scotophase activity ratio.

The activity ratios for the smaller time frames enable a more subtle discrimination of the circadian phenotypes, e.g., the second-to-first half of scotophase activity ratio. In most hamsters, activity started and peaked at the beginning of the scotophase, declined over the course of the scotophase, and had already faded at the beginning of the photophase without clear-cut ending, which could be proven by second-to-first half of scotophase activity ratios smaller 1. SP caused a shift of the hamsters’ activity phase and its peak toward the new beginning of scotophase, which was visualized by actograms and reflected by both activity ratios. This pace enabled defining one slow (hamster #09) and one very slow (hamster #01, “never torpor”) activity responder. During progressing SP adaptation, the actograms of this study suggest increasing flexibility of the daily activity–rest rhythm. This was supported by a decreasing degree of nocturnality, indicated by an increasing photophase-to-scotophase activity ratio. The activity ratios used in this study had no significant influence on and were not influenced by the torpor incidence.

## Conclusion

The incidence, timing, and traits of spontaneous daily torpor show low intra- but high inter-individual variabilities over the torpor period in Djungarian hamsters. Therefore, a detailed body temperature analysis of one representative SP week after complete adaptation might contribute to a refinement of the organ sampling schemes realized in the following week. Hamsters with different torpor incidences, and therefore phenotypes, could then be assigned more equally to different sampling groups regarding metabolic state (hypothermic *vs*. normothermic) at a defined time (torpor entry, deep torpor, and arousal). This standardization will further improve the outcomes of molecular analyses of the torpor regulatory pathways.

Hamsters that never expressed torpor had a low body mass reduction and could be discriminated from torpor-expressing hamsters by high mean body temperatures with low fluctuations in the middle of the scotophase. Moreover, weak or slow SP adaptations of body temperature, activity body mass, and fur index were found. However, an early estimation of the subsequent torpor behavior, and therefore a reduction of animals subjected to implantation, remains difficult because of the high variability of the SP adaptation pace and extent within a hamster cohort. Non-invasive alternative methods like infrared cameras with tracking software to assess activity or indirect calorimetry for metabolic rate measurements should be considered to analyze hamsters’ adaptation and torpor phenotypes before transmitter implantation.

As an immediate response to an abrupt change from LP to SP, scotophase activity and the body temperature decreased in the hamsters, indicating profound physiological changes at the very beginning of adaptation. This underpins the importance of a careful control of the experimental photoperiod regimes and suggests focusing more on the initial metabolic profiles of SP adaptation.

The adaptation parameters gradually changed, even weeks before the anticipated beginning of the energy-demanding winter. This preparatory period allows a fine-tuning of parameters since they start and develop with high variability. The individually fine-tuned set point of body temperature might have a central meaning for torpor integration into the overall energy balance of SP-adapted hamsters.

## Data Availability Statement

The raw data supporting the conclusions of this article will be made available by the authors, without undue reservation.

## Ethics Statement

The animal study was reviewed and approved by Regierungspräsidium Tübingen, Germany (1411).

## Author Contributions

All the authors conceived the project, interpreted the data, and agreed to be accountable for the content of the work. EH and VD performed the experiments and analyzed the data. EH wrote the manuscript, which was carefully revised by all authors.

## Conflict of Interest

The authors declare that the research was conducted in the absence of any commercial or financial relationships that could be construed as a potential conflict of interest.

## Footnotes

1 http://imagej.nih.gov/ij/

2 http://inkscape.org/*developer/

## Supplementary Material

Supplementary Table 1 Background information about the hamsters of this study. The hamsters’ ID comprises the cohorts’ name and a running number. Besides males (m) and females (f), the torpor incidence group is indicated by a raster, from “often torpor” in the first block over “sometimes torpor” and “rarely torpor” to “never torpor” in the last block. The transmitter runtime is given in weeks. The 11 animals of cohort EH04 were implanted already in LP and used in both approaches, while those of the cohorts EH02 and EH03 were implanted when adapted to SP and used in approach 1 only.

Supplementary Table 2 Additional statistics of analysis of approach 1 regarding torpor characteristics and adaptation parameters of the analysis week per torpor incidence group (Tables 1, 2).

Supplementary Table 3 Additional statistics of the radiotelemetry analysis of approach 1 regarding pattern of activity and body temperature of one experimental week per torpor incidence group (Table 3).

Supplementary Table 4 Body temperature values per time frame, week in long photoperiod (LP) and short photoperiod (SP) as well as individual of approach 2. Yellow marked hamsters did not express spontaneous daily torpor. Sparklines in the last column indicate the individual change, with the hamster’s highest value as 100% and its lowest as 0%.

Supplementary Table 5 Delta body temperature values per time frame, week in long photoperiod (LP) and short photoperiod (SP) as well as individual of approach 2. Yellow marked hamsters did not express spontaneous daily torpor. Sparklines in the last column indicate the individual change, with the hamster’s highest value as 100% and its lowest as 0%.

Supplementary Table 6 Activity values per time frame, week in long photoperiod (LP) and short photoperiod (SP) as well as individual of approach 2. Yellow marked hamsters did not express spontaneous daily torpor. Sparklines in the last column indicate the individual change, with the hamster’s highest value as 100% and its lowest as 0%.

Supplementary Table 7 Activity ratio values per time frame, week in long photoperiod (LP) and short photoperiod (SP) as well as individual of approach 2. Yellow marked hamsters did not express spontaneous daily torpor. Red color marks ratios above 1.0 indicating diurnal activity patterns (upper part) or higher activity during the second half of the scotophase (lower part). Sparklines in the last column indicate the individual change, with the hamster’s highest value as 100% and its lowest as 0%. Second to first half of scotophase ratio was not raised for LP with a scotophase of eight hours.

Supplementary Table 8 Adaptation parameter values per week in long photoperiod (LP) and short photoperiod (SP) as well as individual of approach 2. Yellow marked hamsters did not express spontaneous daily torpor. Sparklines in the last column indicate the individual change, with the hamster’s highest value as 100% and its lowest as 0%.

## Abbreviations

HT: hypothermic—body temperature below 32 ° C for more than 30 min, spontaneous daily torpor (on day of sampling)
NT: normothermic—no spontaneous daily torpor (on day of sampling)
LP: long photoperiod—16 h light and 8 h darkness per day
SP: short photoperiod—8 h light and 16 h darkness per day
Std. Dev.: standard deviation of the mean
*T*_b_: core body temperature
ZT: *zeitgeber* time, with ZT0 as the beginning of photophase.
