# Supplementary material for: Body Temperature and Activity Adaptation of Short Photoperiod-Exposed Djungarian Hamsters (Phodopus sungorus): Timing, Traits, and Torpor
Source: Front Physiol. 2021 Jul 7;12:626779. doi: 10.3389/fphys.2021.626779 (PMC8294097; doi:10.3389/fphys.2021.626779)
Supplement: Supplementary Table 1 — Background information about the hamsters of this study. The hamsters’ ID comprises the cohorts’ name and a running number. Besides males (m) and females (f), the torpor incidence group is indicated by a raster, from “often torpor” in the first block over “sometimes torpor” and “rarely torpor” to “never torpor” in the last block. The transmitter runtime is given in weeks. The 11 animals of cohort EH04 were implanted already in LP and used in both approaches, while those of the cohorts EH02 and EH03 were implanted when adapted to SP and used in approach 1 only. [file Data_Sheet_1.docx]

**Supplementary Materials**

Haugg E, Herwig A and Diedrich V (2021) Body Temperature and Activity Adaptation of Short Photoperiod-Exposed Djungarian Hamsters (Phodopus sungorus): Timing, Traits, and Torpor. Front. Physiol. 12:626779.

doi: 10.3389/fphys.2021.626779

Correspondence to: elena.haugg@uni-ulm.de

This file includes:

Supplementary Table 1: Background information about the hamsters (both approaches)

Supplementary Table 2: Additional statistics of torpor characteristics and adaptation parameters (approach 1)

Supplementary Table 3: Additional statistics of the radiotelemetry analysis (approach 1)

Supplementary Table 4: Body temperature values (approach 2)

Supplementary Table 5: Delta body temperature values (approach 2)

Supplementary Table 6: Activity values (approach 2)

Supplementary Table 7: Activity ratio values (approach 2)

Supplementary Table 8: Adaptation parameter values (approach 2)

**SUPPLEMENTS**

Supplementary Table 1: Background information about the hamsters of this study. The hamsters’ ID comprises the cohorts’ name and a running number. Besides males (m) and females (f), the torpor incidence group is indicated by a raster, from “often” torpor in the first block over “sometimes” and “rarely” to “never” torpor in the last block. The transmitter runtime is given in weeks. The eleven animals of cohort EH04 were implanted already in LP and used in both approaches, while those of the cohorts EH02 and EH03 were implanted when adapted to SP and used in approach 1 only.

Supplementary Table 2: Additional statistics of analysis of approach 1 regarding torpor characteristics and adaptation parameters of the analysis week per torpor incidence group (Tables 1 and 2).

Supplementary Table 3: Additional statistics of the radiotelemetry analysis of approach 1 regarding pattern of activity and body temperature of one experimental week per torpor incidence group (Table 3).

Supplementary Table 4: Body temperature values per time frame, week in long photoperiod (LP) and short photoperiod (SP) as well as individual of approach 2. Yellow marked hamsters did not express spontaneous daily torpor. Sparklines in the last column indicate the individual change, with the hamster’s highest value as 100% and its lowest as 0%.

Supplementary Table 5: Delta body temperature values per time frame, week in long photoperiod (LP) and short photoperiod (SP) as well as individual of approach 2. Yellow marked hamsters did not express spontaneous daily torpor. Sparklines in the last column indicate the individual change, with the hamster’s highest value as 100% and its lowest as 0%.

Supplementary Table 6: Activity values per time frame, week in long photoperiod (LP) and short photoperiod (SP) as well as individual of approach 2. Yellow marked hamsters did not express spontaneous daily torpor. Sparklines in the last column indicate the individual change, with the hamster’s highest value as 100% and its lowest as 0%.

Supplementary Table 7: Activity ratio values per time frame, week in long photoperiod (LP) and short photoperiod (SP) as well as individual of approach 2. Yellow marked hamsters did not express spontaneous daily torpor. Red color marks ratios above 1.0 indicating diurnal activity patterns (upper part) or higher activity during the second half of the scotophase (lower part). Sparklines in the last column indicate the individual change, with the hamster’s highest value as 100% and its lowest as 0%. Second to first half of scotophase ratio was not raised for LP with a scotophase of eight hours.

Supplementary Table 8: Adaptation parameter values per week in long photoperiod (LP) and short photoperiod (SP) as well as individual of approach 2. Yellow marked hamsters did not express spontaneous daily torpor. Sparklines in the last column indicate the individual change, with the hamster’s highest value as 100% and its lowest as 0%.
